# Non-volatile metabolomics analysis of heating withering method for processing white tea from Longjing 43 tea variety by UHPLC-Q-TOF/MS

**DOI:** 10.3389/fnut.2025.1735183

**Published:** 2026-01-15

**Authors:** Hongchun Cui, Zhiqiang Cheng, Daliang Shi, Junfeng Yin, Zhihui Feng, Yun Zhao, Jianyong Zhang

**Affiliations:** 1 Tea Research Institute, Hangzhou Academy of Agricultural Science, Hangzhou, China; 2 Tea Research Institute, Chinese Academy of Agricultural Science, Hangzhou, China

**Keywords:** white tea, heating withering, processing technology, taste, quality, mechanism, non-volatile, metabolomics

## Abstract

Withering is a crucial step in the production of white tea. However, the non-volatile metabolomic profile of white tea produced from the Longjing 43 cultivar using heated withering methods has not been fully elucidated. This study investigated the effect of a novel heated withering process on the taste quality and non-volatile metabolome of white tea. The application of heat significantly shortened the withering duration and enhanced the sensory quality of the tea. Specifically, white tea withered using hot air for 23 h, sunlight for 28 h, and fluorescent lamp for 31 h, all at 28 ± 1 °C, achieved higher taste sensory evaluation scores compared to traditional room-temperature withering at 20 ± 1 °C for 40 h. Non-targeted metabolomic analysis led to the identification of 1,372 metabolites across the different withering treatments. Relative to room-temperature withering, the heated withering processes—particularly hot air withering—resulted in higher concentrations of key taste-related compounds, including organic acids, amino acids, flavonoids, and catechins. Enrichment analysis of KEGG pathways indicated that the main metabolic pathways affected by heated withering were cysteine and methionine metabolism, glutathione metabolism, and isoquinoline alkaloid biosynthesis, with the most pronounced effects observed under hot air withering. In summary, hot air withering not only reduced the processing time but also improved the taste quality and markedly altered the profile of key metabolites in white tea.

## 1 Introduction

White tea is one of the six traditional types of tea in China, along with green tea, yellow tea, oolong tea, black tea and dark tea. In terms of processing technique and fermentation degree, it is classified as lightly fermented tea (1–3). Up to now, the development momentum of China’s white tea industry is generally positive. Thirteen provinces—including Hunan, Guizhou, and Guangxi—have established white tea production, with some regions having issued standardized production guidelines (4). In 2025, the domestic sales of white tea in China will approach 11 billion yuan, and the unit price has also shown a significant upward trend (5, 6). These trends clearly reflect the growing attention and notable achievements in the development of the white tea industry. White tea is processed without frying, kneading, or blanching, relying solely on withering and drying (7, 8). The simplest processing procedures create the unique flavor and quality of white tea. Indeed, white tea is widely regarded as one of the least processed tea types, retaining much of its original flavor with minimal human intervention (9, 10). Although the processing technique of white tea is simple, the interaction among amino acids, polyphenols, carbohydrates and other substances in the tea leaves constitutes the unique flavor of white tea (11).

The production of white tea generally consists of two processes: withering and drying, and the key lies in withering. Often described as the “soul” of white tea processing (12), the withering quality largely determines the final product quality (13–15). Withering is a process in which tea leaves undergo slow hydrolysis and oxidation under controlled temperature, humidity, and ventilation, accompanied by water loss and respiration (16). Two main withering methods are traditionally employed: indoor natural withering at room temperature and outdoor solar withering. Outdoor natural withering involves sun-drying tea leaves under ambient weather conditions (17), typically lasting 48 to 72 h (18). Tea masters adjust the process based on experience, observing climatic conditions, leaf moisture loss, color changes, and texture (19). Indoor room-temperature withering, on the other hand, is conducted inside with artificially regulated temperature and humidity, usually spanning 52 to 60 h. Under rainy conditions, indoor withering should not exceed three days, while on sunny and dry days, it should last no less than two days (20). In summary, traditional white tea production predominantly relies on natural withering, which suffers from drawbacks such as long processing time, low efficiency, inconsistent product quality, and high weather dependency. To achieve consistently high-quality white tea, there is an urgent need to adopt novel withering technologies that shorten processing time and improve efficiency.

The withering process gradually dissipates the moisture of fresh tea leaves by changing external environmental factors such as temperature, relative humidity, while internal chemical components transform into flavor-active compounds (21). Both withering temperature and LED light spectrum have been shown to influence respiratory metabolism and the transformation of chemical constituents during this stage (22). Complex biochemical changes occur within the leaves, characterized mainly by a significant decrease in primary catechins, carotenoids, chlorophyll, nucleotides, and nucleosides, alongside a marked increase in free amino acids, monoterpene alcohols, dimeric catechins, and flavonoids (4–6). These changes lay the foundation for the development of white tea’s characteristic quality (23, 24).

A comparative analysis of white tea quality under three withering conditions—indoor natural withering, controlled-temperature machine withering, and outdoor solar withering—revealed that solar-withered tea developed a more distinct floral aroma, while machine-withered tea exhibited a stronger grassy scent. Additionally, ultraviolet radiation during withering promotes the transformation of catechins and enhances tea flavor (25). Different light spectra also significantly affect white tea quality; for instance, red and yellow light withering have been found to improve the sensory quality of Shoumei white tea, with red light yielding the best results. Red light withering significantly increased the content of soluble sugars, amino acids, tea polyphenols, esterified catechins, and total catechin levels compared to solar withering (26). During withering, the concentration of nucleotides increases, while levels of flavonoids and phenolic acids decrease, contributing to the fresh, mellow, and slightly sweet taste of the tea liquor (27). Furthermore, studies have shown that bitter-tasting polyphenols and catechins are catalyzed by polyphenol oxidase to form theaflavins, thearubigins, and theabrownins, while the contents of free amino acids and caffeine increase significantly (28).

In summary, while existing research on white tea has extensively covered aspects such as vintage identification, origin tracing, flavor quality analysis, and technical parameters for withering and drying, the mechanism by which novel heating-assisted withering processes influence the sensory taste quality of white tea and alter non-volatile metabolite profiles remains unclear. This study addresses this gap by comparing the taste and sensory qualities of white tea processed using three heating-assisted withering methods against traditional room temperature withering as a control. Using a multi-dimensional approach—including cluster analysis, principal component analysis, differential metabolite screening, correlation analysis, and KEGG pathway enrichment, we thoroughly investigate how new heating-assisted withering technologies affect taste perception and alter non-volatile metabolite composition. We hope to provide a theoretical basis for understanding and regulating the flavor quality of white tea.

## 2 Materials and methods

### 2.1 Materials and instrument

Fresh tea leaves of the Longjing 43 variety were harvested from Longjing Group Tea Plantation in Xihu District, Hangzhou City, Zhejiang Province. The picking standard is mainly one leaf, three bud, and initial exhibition.

Acetonitrile (chromatographic grade), acetic acid, and ninhydrin were domestically sourced analytical-grade reagents. Methanol, disodium hydrogen phosphate, potassium dihydrogen phosphate, phosphoric acid, sulfuric acid, anthrone, hydrated ninhydrin, and stannous chloride were all domestically produced analytical reagents.

WD05 tea temperature-controlled withering machine (Anxi Yimeng Machinery Co., Ltd., China); 6CLH-60 tea drying machine (Zhejiang Hongwuhuan Tea Equipment Co., Ltd.); LC-30A ultra-performance liquid chromatograph (Shimadzu, Japan); TripleTOF 6600 + mass spectrometer (Foster City, CA, United States); KQ-100E ultrasonic degasser (Jiangsu Kunshan Ultrasonic Instrument Co., Ltd.); 5424R centrifuge (Eppendorf AG, Germany); MU-G02-0448 constant-temperature metal mixer (Hangzhou Mio Instrument Co., Ltd., China); MS105DM electronic balance (Mettler Toledo Instruments Ltd., Switzerland); CentriVap centrifugal concentrator (LABCONCO Ltd., United States).

### 2.2 White tea withering processing method

Fresh leaves of *Camellia sinensis* cv. “Longjing 43” were hand-picked in Hangzhou, China, in May 2025. The tenderness of the fresh tea leaves was one bud with three leaves. White tea processing included the steps of withering and drying.

#### 2.2.1 Method 1 involved the natural withering of white tea under indoor temperature is 20 ± 1 °C (C1)

A total of 5 kg of fresh tea leaves were evenly spread on bamboo trays at an indoor temperature of 20 ± 1 °C and relative humidity of 70–75%, without any auxiliary heating. Withering continued until the leaf moisture content reached 40%, which occurred after 40 h. The withered leaves were then dried using a 6CLH-60 tea dryer: first at 90 °C for 20 min, followed by resting for 1 h, and then a second drying at 50 °C for 20 min. Drying was concluded when the moisture content fell below 6%, yielding the finished white tea sample (C1).

#### 2.2.2 Method 2 involved the heating withering of white tea under hot air temperature 28 ± 1 °C (C2)

Five kilograms of fresh leaves were withered using a WD05 temperature-controlled withering machine, which maintained a hot air temperature of 28 ± 1 °C and relative humidity of 70–75%. Withering was complete when the moisture content reached 40%, requiring 23 h in total. The subsequent drying procedure was identical to that described in Method 1, resulting in sample C2.

#### 2.2.3 Method 3 involved the heating withering of white tea under sunlight temperature 28 ± 1 °C (C3)

Five kilograms of fresh leaves were withered outdoors under natural sunlight, at an ambient temperature of 28 ± 1 °C and relative humidity of 70–75%. This process concluded after 28 h upon reaching the target moisture content of 40%. Drying was then performed as in Method 1, producing sample C3.

#### 2.2.4 Method 4 involved the heating withering of white tea under fluorescent lamp temperature 28 ± 1 °C (C4)

For this treatment, 5 kg of fresh leaves were withered indoors under fluorescent lamp irradiation, with the environment maintained at 28 ± 1 °C and 70–75% relative humidity. The withering endpoint (40% moisture content) was achieved after 31 h. The leaves were then dried using the same protocol as the other methods, yielding sample C4.

### 2.3 Sample preparation for metabolomic analysis

Finished white tea samples from each withering treatment were freeze-dried (Scientz-100F lyophilizer), ground into a fine powder (MM 400 grinder, 30 Hz, 1.5 min), and then 30 mg of the powder was accurately weighed. The powder was extracted with 1,500 μL of a pre-cooled (−20 °C) 70% methanol aqueous solution containing internal standards. The mixture was vortexed every 30 min for 30 s, repeated six times. After centrifugation (rotation speed 12,000 rpm, 3 min), the supernatant was aspirated. The sample was filtered through a microporous membrane (0.22 μm pore size) and stored in the injection vial for UPLC-MS/MS analysis. Quality control (QC) samples were prepared by combining equal aliquots from all individual samples.

### 2.4 Sensory evaluation of white tea with different withering process

The taste quality of white tea samples was assessed according to standard sensory evaluation methods. Briefly, 3 g of each tea sample was brewed with 100 mL of boiling water in a lidded tasting cup for 5 min. The resulting tea infusion was then transferred to a tasting bowl. The taste attributes and score of the tea infusions samples were described by 8 reviewers (four males and four females, aged 25–38 years). A percentage scale was adopted by taste sensory quantitative evaluation.

### 2.5 UHPLC-Q-TOF/MS metabolomic analysis

Non-targeted metabolomic profiling of the white tea samples was performed using an LC-30A UPLC system (Shimadzu, Japan) coupled with a TripleTOF 6600 + mass spectrometer (Foster City, CA, United States). All samples were acquired by the LC-MS system followed machine orders. The Waters ACQUITY UPLC HSS T3 (1.8 μm, 2.1 mm × 100 mm) was used at a column temperature of 40 °C. The flow rate was set as 0.40 mL/min. The injection volume was 4 μL. The mobile phase A was water (0.1% formic acid). The mobile phase B was acetonitrile (0.1% formic acid). The quantitative analysis of the sample was as follows: from 0 to 5 min, mobile phase B increased from 5 to 65%; from 5 to 6 min, mobile phase B increased to 99%; from 6 to 7.5 min, mobile phase B kept for 99%; from 7.5 to 7.6 min, mobile phase B decreased from 99 to 5%; from 7.6 to 10 min, mobile phase B kept for 5%.

The information-dependent acquisition (IDA) mode of Analyst TF 1.7.1 Software (Sciex, Concord, ON, Canada) were employed to operate data acquisition. The source parameters were set as follows: ion source gas 1 (GAS1), 50 psi; ion source gas 2 (GAS2), 60 psi; curtain gas (CUR), 35 psi; temperature (TEM), 550 °C, or 550 °C; declustering potential (DP), 80 V, or −80 V in positive or negative modes, respectively; and ion spray voltagefloating (ISVF), 5,500 V or −4,500 V in positive or negative modes, respectively. The TOF MS scan parameters were set as follows: mass range, 50–1,250 Da; accumulation time, 200 ms; and dynamic background subtract, on. The product ion scan parameters were set as follows: mass range, 50–1,250 Da; accumulation time, 40 ms; collision energy, 30 or −30 V in positive or negative modes, respectively; collision energy spread, 15; resolution, UNIT; charge state, 1 to 1; intensity, 100 cps; exclude isotopes within 4 Da; mass tolerance, 50 mDa; maximum number of candidate ions to monitor per cycle, 12.

The relative areas were employed in subsequent statistical analyses. The original data of mass spectrometry was converted into mzML format by proteowizard, and the XCMS program was used for peak extraction, alignment and retention time correction. The peaks with missing rate >50% in each group were filtered, and the blank value was filled with KNN +1/5 minimum value (the blank value >50% was filled with 1/5 minimum value, and the blank value <50% was filled with KNN), and the peak area was corrected by SVR method. After calibration and screening, the metabolites were identified by searching the self built database, integrating the public library, prediction library method. Finally, the substances with a comprehensive identification score of more than 0.5 and a CV value of QC samples less than 0.5 were extracted, and then the positive and negative modes were combined (the substances with the highest qualitative grade and the lowest CV value were retained), and the differential metabolites were obtained by analysis.

### 2.6 Statistical analysis

Metabolomics data were analyzed using the following statistical methods. Unsupervised principal component analysis (PCA) was performed using the prcomp function in R, with data preprocessed by unit variance scaling. Hierarchical cluster analysis (HCA) and Pearson correlation analysis were conducted using the Complex Heatmap package in R. HCA results were displayed as heatmaps with dendrograms, while correlation patterns between samples were visualized using heatmaps based on Pearson correlation coefficients (PCC). Metabolite intensities were normalized by unit variance scaling prior to HCA.

Differential metabolites between two groups were identified based on variable importance in projection (VIP >1) from OPLS-DA models and absolute log2-fold change (|Log₂FC| ≥1.0). OPLS-DA was performed using the MetaboAnalystR package after log2 transformation and mean-centering of the data. A permutation test (200 iterations) was applied to prevent overfitting.

Metabolites were annotated against the KEGG compound database and subsequently mapped to KEGG Pathway databases for enrichment analysis. Statistical analyses, including ANOVA and multiple comparisons, were performed using SPSS 25. All graphs were generated using Excel 2023 and Origin 2021, with three biological replicates per sample.

## 3 Results

### 3.1 Sensory quality characteristics of white tea with different heating withering process

**Figure 1:**
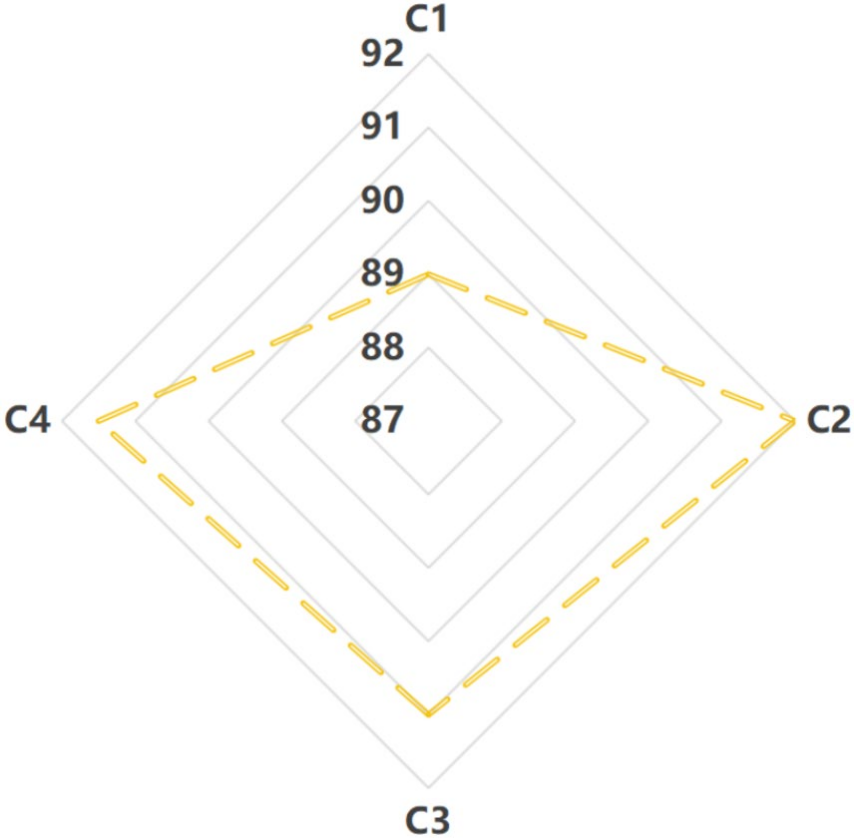
Taste sensory quality of four white tea samples with different withering processes. White tea with natural traditional room temperature withering in 20 ± 1 °C (C1); white tea with hot air heating withering in 28 ± 1 °C (C2); white tea with sunlight heating withering in 28 ± 1 °C (C3); white tea with fluorescent lamp heating withering in 28 ± 1 °C (C4).

The sensory evaluation results of different withering treatments for white tea taste are shown in Figure 1. Compared to the control (C1, traditional room-temperature withering), the taste sensory scores of white tea processed by hot air (C2), sunlight (C3), and fluorescent lamp (C4) heating withering were all higher. The taste scores of white tea by three heating withering processes from high to low are C2 > C4 > C3. The taste sensory evaluation description of white tea by traditional room temperature withering process (C1) was weak sweetness, weak mellow and mild, slightly green. In contrast, the C2 sample was described as having very strong sweetness and high mellowness. The C3 sample exhibited relatively strong sweetness and richness, while the C4 sample showed moderate sweetness and mellowness. Overall, the hot air-withered white tea (C2) scored highest in clarity, sweetness, and richness, indicating superior taste quality, followed by C4 and then C3. Li investigated the effects of room-temperature withering time, temperature, and drying processes on the composition and quality of Fuding Da Bai tea, reporting optimal results with a 28 h natural withering time and 95 °C drying temperature (8). However, the tea cultivar and room-temperature withering conditions in that study differed from those used here, which may account for the differing outcomes.

### 3.2 Overall analysis of the metabolic profile of white tea by the heating withering process

**Figure 2:**
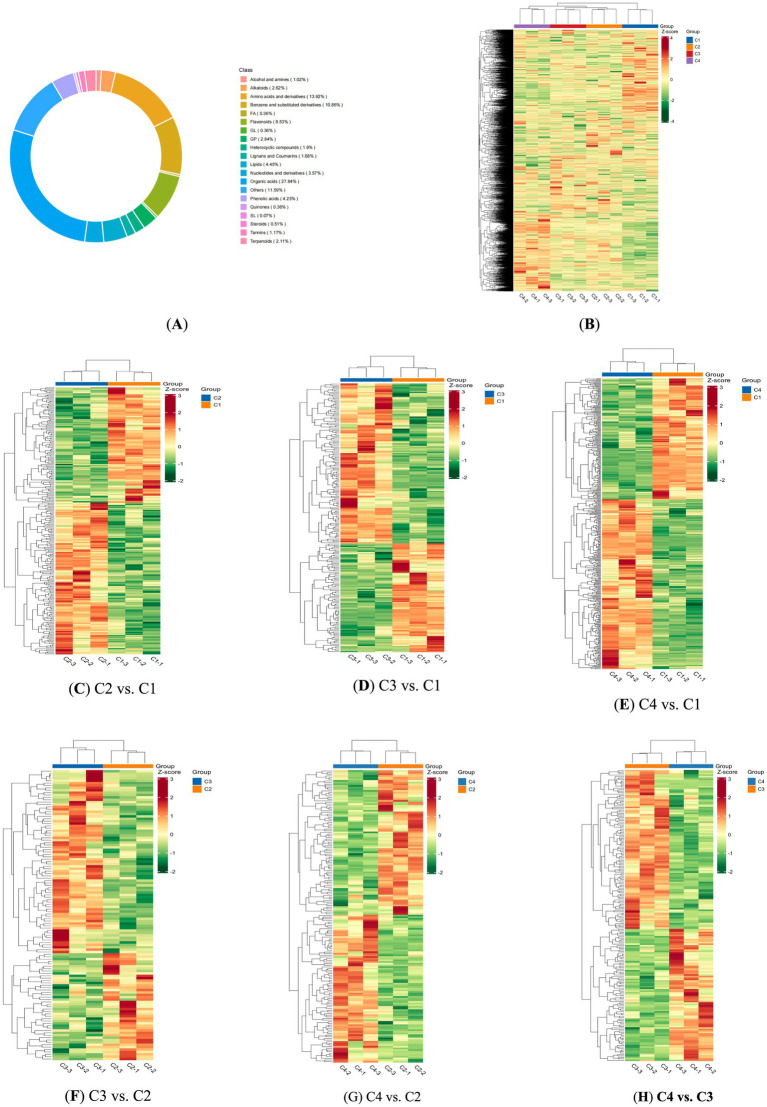
Heat maps of four white tea samples with different withering processes. **(A)** A heat map overview of the non-volatile components of the four withering processes. **(B)** Metabolite categories form a circular diagram. **(C)** Heat map analysis of non-volatile components in C2 vs. C1. **(D)** Heat map analysis of non-volatile components in C3 vs. C1. **(E)** Heat map analysis of non-volatile components in C4 vs. C1 **(F)** Heat map analysis of non-volatile components in C3 vs. A2. **(G)** Heat map analysis of non-volatile components in C4 vs. C2. **(H)** Heat map analysis of non-volatile components in C4 vs. C3.

Cluster Analysis was a classified multivariate statistical analysis method (25). Classify individuals or samples based on their characteristics, ensuring that individuals within the same category have as high homogeneity as possible, while categories should have as high heterogeneity as possible. Cluster analysis was employed to overall analysis the metabolic profile of white tea by the heating withering process. Through the analysis of the proportion of metabolite composition, the distribution of major metabolites in white tea under different withering treatments could be examined as a whole (Figure 2A).

A total of 1,372 metabolites of white tea under different withering processes were identified, belonging to various classes, including 14 alcohol and amines, 36 alkaloids, 191 amino acids and derivatives, 149 benzene and substituted derivatives, 5 free fatty acids, 117 flavonoids, 5 glycerides, 39 glycerol phospholipids, 26 heterocyclic compounds, 23 lignans and coumarins, 61 lipids, 49 nucleotides and derivatives, 382 organic acids, 159 others (fatty acylates), 58 phenolic acids, 5 quinones, 1 sphingolipids, 7 steroids, 16 tannins, 29 terpenoidsb (Figure 2B).

Compared to C1, 136 metabolites showed higher abundance in C2 (Figure 2C), including Leu-Phe-Ile-Asp, atalaphylline, His-Val-Leu-Lys, Thr-Tyr-Arg-Lys, Thr-Arg-Gln-Glu, glutamyl-aspartyl-glycine, epigallocatechin gallate, catechin, gallocatechin, myricetin 3-O-β-D-galactopyranoside, kaempferol 3-O-β-D-glucopyranosyl-(1 → 3)-α-L-rhamnopyranosyl-(1 → 6)-β-D-galactopyranoside, quinaldic acid, oxidane sulfonic acid, Asp-Pro-Arg, Trp-Asp sulfonic acid, mallotophenone, His-Leu-Val-Leu-Arg, ochrolifuanine A, 3-Hydroxyflavone, toyocamycin, Val-Ala-Gly-Val-Ala, Arg-Ala-Leu-Lys, lysyl-alanyl-alanine, pimpinellin, vialinin A, thiobinupharidine, aloeresin A, hecogenin, (+)-catechin gallate, leucode lphidin, sativoside, thalidasine, echinocandin B, acteoside, palmyramide A etc. The taste sensory quality of C2 was higher than that of C1, which might be closely related to these metabolic substances.

Relative to C1, 124 metabolites were more abundant in C3 (Figure 2D), such as catechin gallate, myricetin-3-O-α-L-rhamnopyranosyl-(1 → 6)-β-D-glucopyranoside, quercetin 3-O-α-L-rhamnopyranosyl-(1 → 3)-α-L-rhamnopyranosyl-(1 → 6)-β-D-galactopyranoside, 4-hydroperoxy-2-nonenal, sulfonic acid, 6′,7′,10,11-tetramethoxy-8-Desoxygartanin, Trp-Ala-Tyr, trans-2-Octenoic acid, 1-Heptadecanoyl-sn-glycero-3-phosphocholine, thalidasine, madecassic acid, 1-palmitoyl-2-linoleoyl-sn-glycero-3-phosphate, 6-ethylmercaptopurine, myricitrin, Ala-Leu-Cys, 3,4,5-trihydroxyoxane-2-carboxylic acid, epicatechin gallate, epicatechin, 9-cis-Retinol, Arg-Val-Asn-His-Val, ercalcitriol, cyclopassifloside, betulonic acid, canthiumine, nervonic acid, acteoside, His-Val-Leu-Lys, 4-methylumbelliferyl acetate, Val-His-His, carnitine-2-methyl-C4, Lys-His-Ile-Leu-Ala, sulfazecin, His-His-Val-Ala-Tyr, spiramycin, ochrolifuanine A, prilocaine, Tyr-Ser-Asn-Arg, His-Tyr-Arg, vialinin A, dihydrorobinetin, mallotinic acid, phe-Met-Val, His-Leu-Val-Leu-Arg, muscarine, Cys-Trp-Ser, Arg-Ser-Lys-Arg, 3,4,5-trihydroxyoxane-2-carboxylic acid, triptophenolide, etc. The taste sensory quality of C3 was higher than that of C1, which might be closely related to these metabolic substances.

Similarly, 248 metabolites were upregulated in C4 compared to C1 (Figure 2E), including mallotophenone, 9-oxo-nonanoic acid, 13-oxo-9,11-octadecadienoic acid, epigallocatechin gallate, catechin, gallocatechin, kaempferol 3-O-β-D-glucopyranosyl-(1 → 3)-α-L-rhamnopyranosyl-(1 → 6)-β-D-glucopyranoside, K-ga-r-r, kaempferol 3-O-α-L-rhamnopyranosyl-(1 → 3)-α-L-rhamnopyranosyl- (1 → 6)-β-D-galactopyranoside, cyclopassifloside, lrisxanthone, thalidasine, 1,2-Dipalmitoyl-sn-glycerol, 3,4,5-trihydroxyoxane-2-carboxylic acid, phloretin-4′-O-(6″-Salicyloyl)glucoside, Thr-Met-Leu, Ser-Gln-Leu-Lys, triptophenolide, sulfonic acid, 11-(3,4-dimethyl-5-propylfuran-2-yl)undecanoate, Hexadecanedioic acid, glyceryl arachidonate, N-cyclohexyl-4-(dimethylamino)benzenecarbothioamide, Asn-Ala-Leu-Ala-His, 14-methylpentadecanoate, isosinomenine, eplerenone, Thr-Tyr-Arg-Lys, Isomaltose, sativoside, H-Leu-Trp-OH, phosphonoacetate, dTDP-beta-L-mycarose, Thr-Arg-Gln-Glu, palmyramide A, TyrMe-Nap-OH, Tyr-Pro-Phe, Arg-Val-Asn-His-Val, Arg-Tyr-Val-Lys, undecanoic acid, dihydrorobinetin, sulfazecin, Lys-Cys-Asp-Pro-Thr, His-Leu-Val-Leu-Arg, Ile-Arg-Tyr-Arg, jasmone, geranyl acetate, hecogenin, ostruthin, trigonelline, betulin, Ala-His-Tyr-Glu, thiobinupharidine etc. The taste sensory quality of C4 was higher than that of C1, which might be closely related to these metabolic substances.

From the above results, it could be seen that the levels of organic acids, amino acids, flavonoids, and catechins content in C2, C3, C4 were relatively higher than in C1, which providing a chemical basis for their improved taste quality.

The superior taste of C3 over C2 suggested distinct metabolite profiles. Indeed, 66 metabolites were more abundant in C2 than in C3 (Figure 2F), including N-acetylsphinganine, Leu-Asp-Gln, 2-Octyl-1-dodecanol, leucodelphidin, pimpinellin, 3-Hydroxyflavone, methyl 3,15,21-tris[[3,4,5-trihydroxy-6-(hydroxymethyl)oxan-2-yl]oxy], epicatechin-3-O-gallate, docosanoate, 2,4,6-Trihydroxybenzophenone, 4,5-Dihydroxyphthalic acid, toyocamycin, docosylacetate, Ethanolamide, goyaglycoside c, 1-Palmitylthio-2-palmitoylamido-1,2-dideoxy-sn-glycero-3-phosphorylcholine, gossypetin 3,3’-dimethyl ether, kaempferol3-rhamninoside, digoxigenin, dioleoyl phosphatidylserine, N-Acetylputrescine, heneicosanoic acid.

The superior taste of C2 over C4 suggested distinct metabolite profiles. Indeed, 66 metabolites were more abundant in C2 than in C4 (Figure 2G), including epicatechin, epigallocatechin gallate, gallocatechin gallate, quercetin 3-O-α-L-rhamnopyranosyl-(1 → 6)-β-D-galactopyranoside; Q-g-r,quercetin 3-O-α-L-rhamnopyranosyl-(1 → 6)-β-D-glucopyranoside, tiapamil, H-Leu-leu-phe-OH, Lys-Ser-Asp-Glu-Thr, oxeladin, (2-Oxo-1,2-diphenylethoxy)sulfonic acid, Thr-Pro-Tyr, Flavone, sulfonic acid, diphthamide, Lys-Tyr-Thr-Arg, tricoumaroyl spermidine, caffeic acid phenethyl ester, butanoic acid, Val-Cys-Ser, guanosine-5′-monophosphate, benzoic acid, 3,4,5-trihydroxyoxane-2-carboxylic acid, Ala-Phe-Gln-Lys, Glu-Phe-Thr-Asp, Met-Ser-Tyr, Met-Val-His, Cys-Trp-Ser, cannabisin A, 3,5,7,3′,5′-Pentahydroxyflavan, phenylbutazone, glutathione reduced form, Ser-Ile-Gly-Ser-Leu-Ala-Lys, Trp-Glu-Leu, oxidanesulfonic acid, Cys-Phe-Glu, oxane-2-carboxylic acid, sulfonic acid, pantetheine, isobutyraldehyde oxime, baicalin, 2-hexyldecanoic acid, 5-acetylthiophene-2-carboxylic acid, octadecadienamide, acadesine, 3-oxovalproic acid, manniflavanone, 2-alpha-linolenoyl-glycerol, aralionine A etc. From the above results, it could be seen that the amino acids, flavonoids content in C2 were relatively higher than C4, which might be the chemical basis for the superior taste quality of the hot air heating withering process compared to the fluorescent lamp heating withering process.

Similarly, the taste superiority of C4 over C3 correlated with metabolic differences. Seventy-nine metabolites were more abundant in C4 than in C3 (Figure 2H), including epicatechin gallate, quercetin 3-O-β-D-glucopyranosyl-(1 → 3)-α-L-rhamnosyl-(1 → 6)-β-D-galactopyranoside, 9-oxo-nonanoic acid, oglufanide, thalidasine, pinolenic acid, pomalidomide, oxidanesulfonic acid, tetracosanal, Thr-Met-Leu, isomaltose, Leu-Tyr-Phe-Lys, Leu-Asp-Gly, catechin, strophanthidin 3-diglucosylcymarose, Arg-Tyr-Val-Lys, Val-Gly-Lys-Lys-Gln, Pro-Lys-His, 3,4,5-trihydroxyoxane-2-carboxylic acid, spiramycin, thiobinupharidine, 8-demethyl-8-(dimethylamino)riboflavin, cercosporin, protocetraric acid, leucodelphidin, isolimocitrol, papaverine, S-(1,2-Dicarboxyethyl)glutathione, threonine, lysylglutamic acid, benzimidazole, cellobiotol, 1-Decanoyl-sn-glycero-3-phosphocholine, geranyl diphosphate, digitoxin, mulberrin etc. From the above results, it could be seen that the amino acids, flavonoids content in C4 were relatively higher than C3, which might be the chemical basis for the superior taste quality of the fluorescent lamp heating withering process compared to the sunlight heating withering process.

The withering process of white tea is the first step in the processing of white tea and the key basic process for the formation of white tea flavor and quality. Han compared the flavor and quality characteristics of mulberry leaf white tea when naturally withered indoors (25 °C) and heated in an oven (30 °C), and found that revealing significant differences in the levels of leachate, tea polyphenols, DNJ, and GABA (3).

### 3.3 Analysis of differential metabolites in heated withering process based on PCA

Principal component analysis (PCA) was a multi-dimensional data statistical analysis method for unsupervised pattern recognition. Principal component analysis (PCA) was employed to analysis the differential metabolites in heated withering process. PC1 represented the first principal component. PC2 represented the second principal component. The percentage indicated the interpretation rate of this principal component for the datasets. The values of the main components PC1 and PC2 indicated the degree of change in the content of metabolites in white tea processed by different withering techniques in the corresponding directions. Smaller PC1 and PC2 values usually indicated that the variation range of metabolite content in white tea processed by different withering techniques in this direction was relatively small.

**Figure 3:**
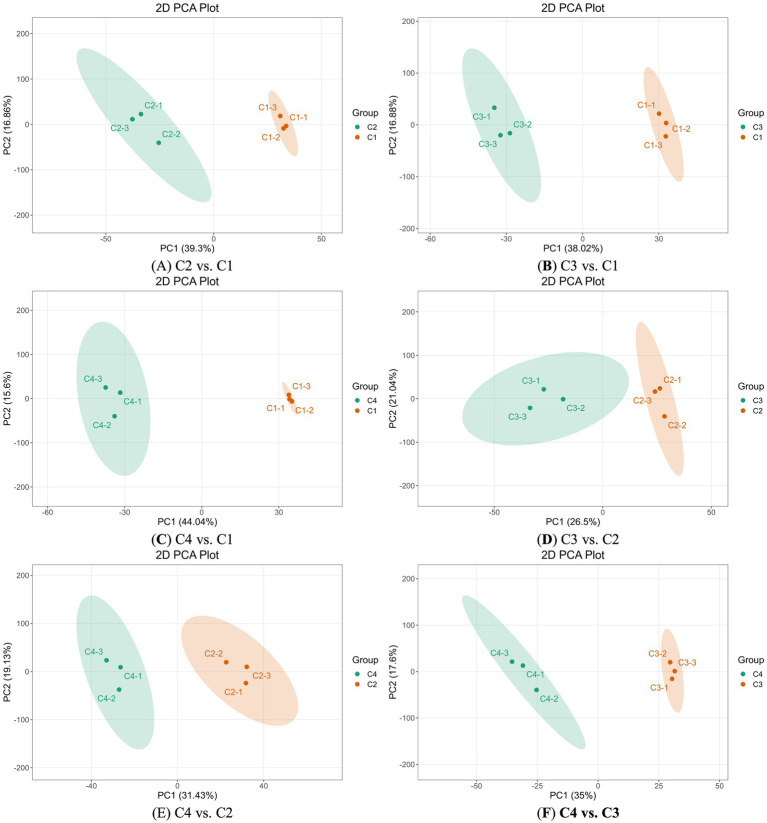
PCA analysis of four white tea samples with different withering processes. Each point in the figure represented a sample. Samples in the same group were indicated by the same color. **(A)** PCA analysis of non-volatile components in C2 vs. C1. **(B)** PCA analysis of non-volatile components in C3 vs. C1. **(C)** PCA analysis of non-volatile components in C4 vs. C1 **(D)** PCA analysis of non-volatile components in C3 vs. A2. **(E)** PCA analysis of non-volatile components in C4 vs. C2. **(F)** PCA analysis of non-volatile components in C4 vs. C3.

The variation degree of the main component content of the metabolites of heated withering white tea with that of the control indoor naturally withering white tea was compared. From Figures 3A–C, the PC1 of C2 and C1, C3 and C1, C4 and C1 was 39.3, 38.02, 44.04%, respectively, indicating that the variation range of metabolite content of C3 and C1 was relatively small based on PC1. The PC2 of C2 and C1, C3 and C1, C4 and C1 was 16.86, 16.88, 15.6%, respectively, indicating that the variation range of metabolite content of C4 and C1 was relatively small based on PC2. The variability degree of metabolite content in white tea processed by hot air heating withering (C2) and natural indoor withering (C1) in this dimension was relatively high, consistence with the situation where there was the largest relative difference in taste sensory evaluation scores between C2 and C1.

The variation degree of metabolic main components content of white tea under different heating withering processes were compared. From Figures 3D–F, the PC1 of C3 and C2, C4 and C2, C4 and C3 was 26.5, 31.43, 35%, respectively, indicating that the variation range of metabolite content of C3 and C2 was relatively small based on PC1. The PC2 of C3 and C2, C4 and C2, C4 and C3 was 21.04, 19.13, 17.6%, respectively, indicating that the variation range of metabolite content of C4 and C3 was relatively small based on PC2. The trend of variation degree in metabolite content under different heating withering processes did not correspond to the trend of changes in those taste sensory quality scores. The regulation of taste metabolites and quality of white tea by heating withering was quite complex.

### 3.4 Analysis of differential metabolites in heated withering process based on OPLS-DA

**Figure 4:**
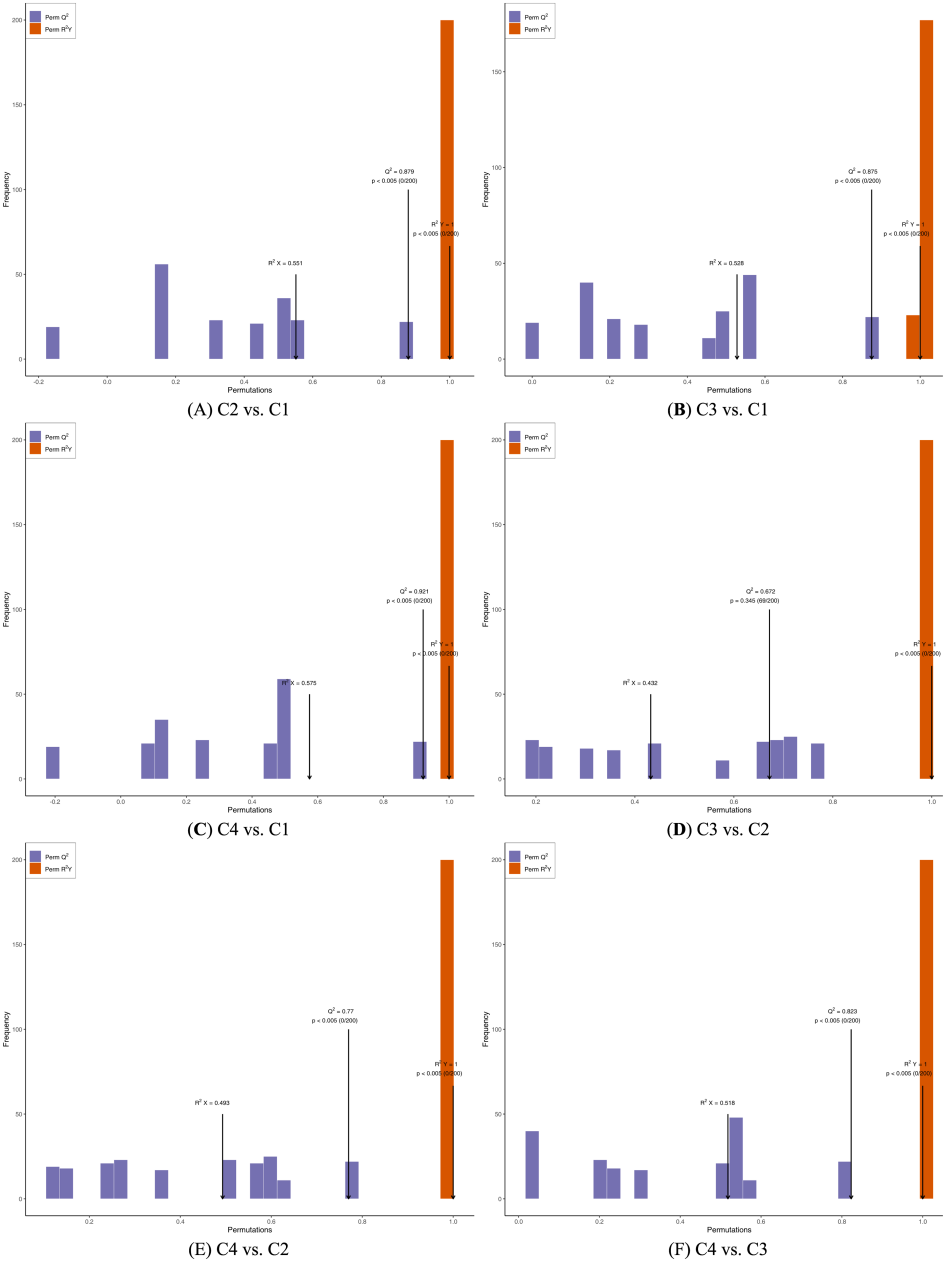
OPLS-DA analysis of four white tea samples with different withering processes. **(A)** OPLS-DA analysis of non-volatile components in C2 vs. C1. **(B)** OPLS-DA analysis of non-volatile components in C3 vs. C1. **(C)** OPLS-DA analysis of non-volatile components in C4 vs. C1. **(D)** OPLS-DA analysis of non-volatile components in C3 vs. A2. **(E)** OPLS-DA analysis of non-volatile components in C4 vs. C2. **(F)** OPLS-DA analysis of non-volatile components in C4 vs. C3.

Orthogonal projections to latent structures-discriminant analysis (OPLS-DA) was a multivariate statistical analysis method for supervised pattern recognition. The specific approach was to extract the components in the independent variable *X* and the dependent variable *Y* respectively, and then calculate the correlation between the components (29). Compared with PCA, OPLS-DA can maximize the differentiation between groups and is conducive to finding differential metabolites. Orthogonal partial least squares discriminant analysis (OPLS-DA) combined orthogonal signal correction (OSC) and OPLS-DA methods. It could decompose the information of the *X* matrix into two types of information related to *Y* and unrelated, and screened the differential variables by eliminating the unrelated differences. The prediction parameters of the OPLS-DA evaluation model were *R*^2^*X*, *R*^2^*Y* and *Q*^2^. Among them, *R*^2^*X* and *R*^2^*Y* represented the interpretation rates of the established model for the *X* and *Y* matrices respectively, and *Q*^2^ represents the prediction ability of the model. The closer these three indicators were to 1, the more stable and reliable the model is. When *Q*^2^ > 0.5, it could be regarded as a valid model. When *Q*^2^ > 0.9, it was an excellent model. The Figure 4 was the verification diagram of OPLS-DA.

The predictability (*Q*^2^) and goodness of fit (*R*^2^) of OPLS-DA models were observed for comparisons between C2 and C1 (*Q*^2^ = 0.879, *R*^2^*X* = 0.551, *R*^2^*Y* = 1, Figure 4A), as well as between C3 and C1 (*Q*^2^ = 0.875, *R*^2^*X* = 0.528, *R*^2^*Y* = 1, Figure 4B), C4 and C1 (*Q*^2^ = 0.921, *R*^2^*X* = 0.575, *R*^2^*Y* = 1, Figure 4C), C3 and C2 (*Q*^2^ = 0.672, *R*^2^*X* = 0.432, *R*^2^*Y* = 1, Figure 4D), C4 and C2 (*Q*^2^ = 0.77, *R*^2^*X* = 0.493, *R*^2^*Y* = 1, Figure 4E), and C4 and C3 (*Q*^2^ = 0.823, *R*^2^*X* = 0.518, *R*^2^*Y* = 1, Figure 4F). In the OPLS-DA models, The *p* value of all OPLS-DA evaluation models was less than 0.005, indicating that the metabolite analysis models of different withering white tea processing techniques were better (Figure 3). The p of the OPLS-DA evaluation models of the six withering processes of white tea was all less than 0.005, indicating that the metabolite analysis models of different withering processes of white tea were better.

### 3.5 Screening of differential metabolites in white tea processed by heated withering

Based on the variable importance in projection (VIP) obtained from the OPLS-DA model, metabolites with differences among different withering process groups could be preliminarily screened out. At the same time, the *p*-value/FDR (biological repeat ≥2) or FC value of univariate analysis could be combined to further screen out the differential metabolites of differential withering processes (30). The screening criteria for differential metabolites of white tea with different withering processes in this study were VIP >1, fold change ≥2 and fold change ≤0.5 VIP >1.

**Figure 5:**
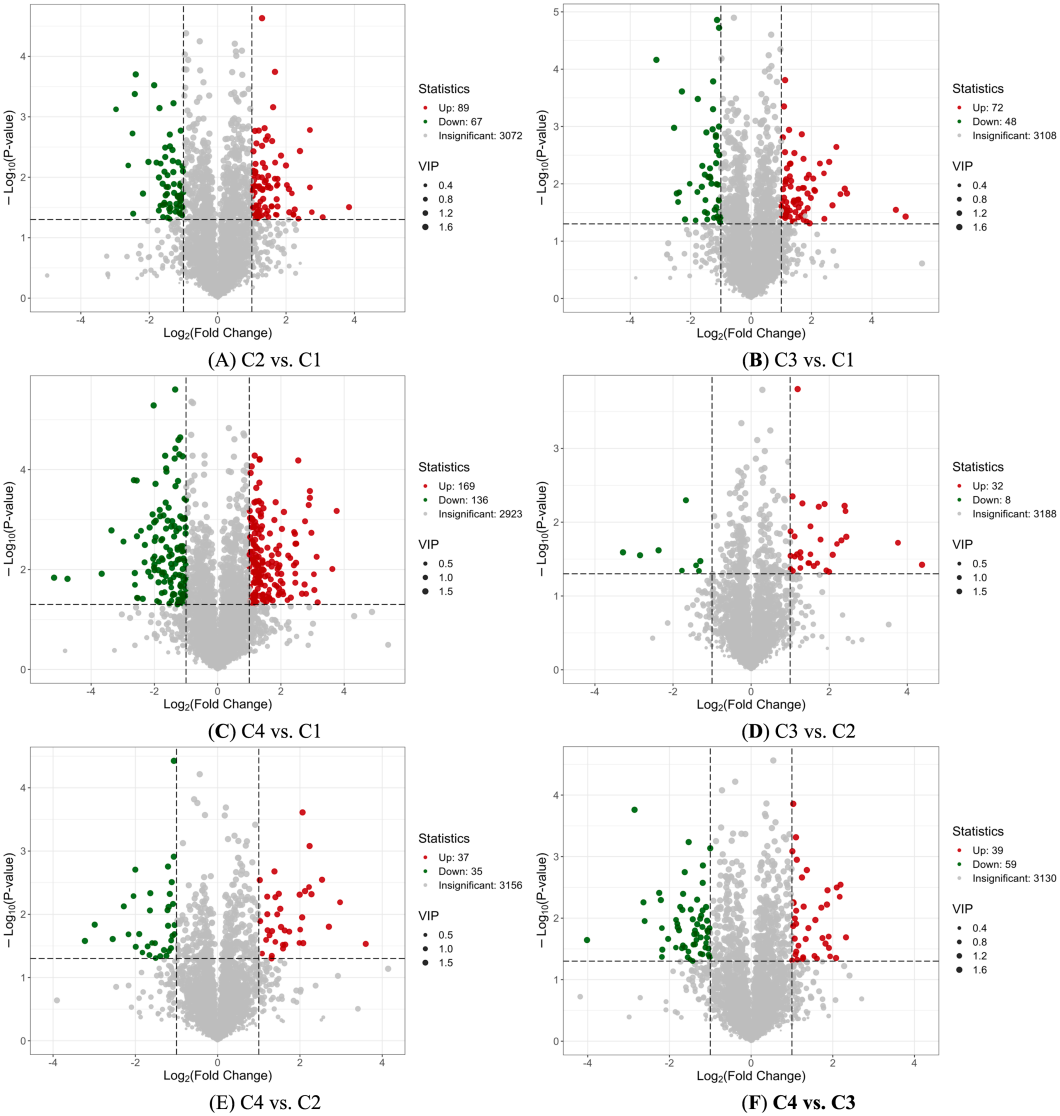
Volcano plot analysis of four white tea samples with different withering processes. **(A)** Volcano plot analysis of non-volatile components in C2 vs. C1. **(B)** Volcano plot analysis of non-volatile components in C3 vs. C1. **(C)** Volcano plot analysis of non-volatile components in C4 vs. C1 **(D)** Volcano plot analysis of non-volatile components in C3 vs. A2. **(E)** Volcano plot analysis of non-volatile components in C4 vs. C2. **(F)** Volcano plot analysis of non-volatile components in C4 vs. C3. Each point in the volcano map represented a metabolite. The green points represented the down-regulated differential metabolites. The red points represent the up-regulated differential metabolites. The gray points represented the metabolites detected but with insignificant differences. The horizontal axis represented the logarithm of the multiple of the relative content difference of a certain metabolite in the two groups of samples (Log_2_FC). The vertical axis represented the level of significance of the difference (−Log_10_ *p*-value). The size of the dot represents the VIP value.

The volcano plot was used to show the differences in the relative content of metabolites in white tea processed by different withering techniques between two groups of samples and the significance of the statistical differences. There were 89 up-regulated metabolites and 67 down-regulated metabolites between C2 and C1 (Figure 5A); 72 up-regulated metabolites and 48 down-regulated metabolites between C3 and C1 (Figure 5B); and 169 up-regulated and 136 down-regulated between C4 and C1 (Figure 5C). In a word, comparing with the metabolites of C1 traditional room temperature withering process control, there were differences in the up-regulation and down-regulation of metabolites in different heating withering processes, especially in the fluorescent lamp withering process (C4).

The differences in up-regulated and down-regulated metabolites among white tea samples subjected to different heating and withering processes were compared. There were 32 up-regulated metabolites and 8 down-regulated metabolites between C3 and C2 (Figure 5D); 37 up-regulated metabolites and 35 down-regulated metabolites between C4 and C2 (Figure 5E); and 39 up-regulated and 59 down-regulated between C4 and C3 (Figure 5F). Unlike the upregulation and downregulation of metabolites in the aforementioned traditional room temperature withering and heated withering of white tea, the number of differences in upregulation and downregulation of metabolites among white tea samples subjected to different heated withering processes were significantly smaller. The treatment with relatively large amounts of up-regulated and down-regulated metabolites was sunlight heating withering (C3) and fluorescent lamp heating withering (C4).

**Figure 6:**
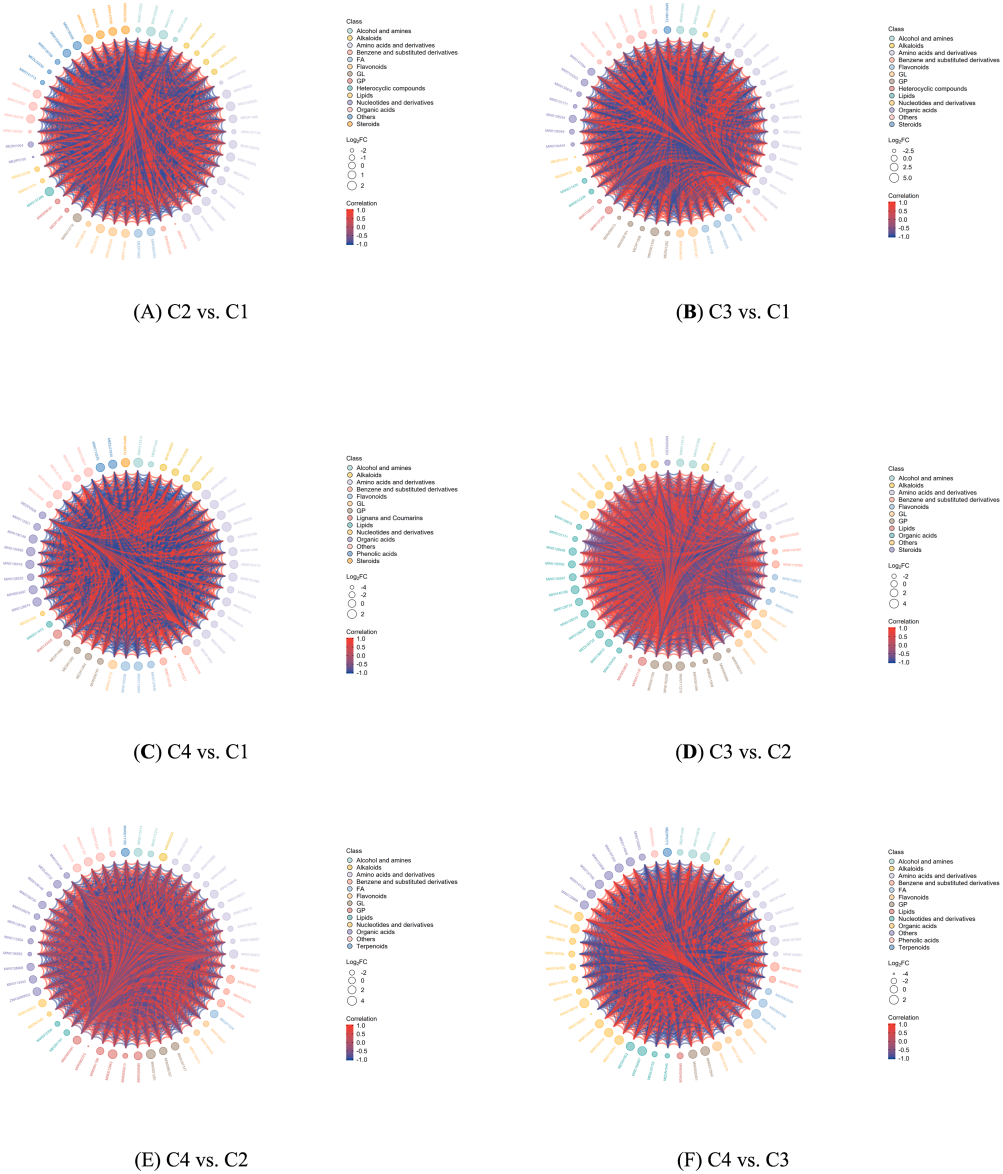
Metabolite association analysis of four white tea samples with different withering processes. **(A)** Metabolite association analysis of non-volatile components in C2 vs. C1. **(B)** Metabolite association analysis of non-volatile components in C3 vs. C1. **(C)** Metabolite association analysis of non-volatile components in C4 vs. C1 **(D)** Metabolite association analysis of non-volatile components in C3 vs. A2. **(E)** Metabolite association analysis of non-volatile components in C4 vs. C2. **(F)** Metabolite association analysis of non-volatile components in C4 vs. C3. The outermost layer in the figure represented the names of the differential metabolites. The size of the dots indicated the Log_2_FC value of the corresponding differential metabolite. Different colors represented different classifications (classes) corresponding to different metabolites. The lines connecting represented the magnitudes of the Pearson correlation coefficients between the corresponding differentially expressed metabolites. Red lines represented positive correlations, while blue lines represented negative correlations.

There was a synergistic or mutually exclusive relationship among the metabolites of white tea processed by different withering techniques. Correlation analysis could help measure the metabolic closeness among the metabolites with significant differences, which was conducive to further understanding the mutual regulatory relationship among the metabolites during the state changes of tea processed by withering techniques. The Pearson correlation analysis method was used to conduct a correlation analysis on the differential metabolites of white tea with different withering processes identified according to the screening criteria. The number of differential metabolites with a correlation coefficient greater than or equal to 1 between C2 and C1 was 57 (Figure 6A). The number of differential metabolites with a correlation coefficient greater than or equal to 1 between C3 and C1 was 50 (Figure 6B). The number of differential metabolites with a correlation coefficient greater than or equal to 1 between C4 and C1 was 50 (Figure 6C). The number of differential metabolites with a correlation coefficient greater than or equal to 1 between C3 and C2 was 51 (Figure 6D). The number of differential metabolites with a correlation coefficient greater than or equal to 1 between C4 and C2 was 50 (Figure 6E). The number of differential metabolites with a correlation coefficient greater than or equal to 1 between C4 and C3 was 51 (Figure 6F).

### 3.6 Analysis of the differential metabolic pathways of flavor substances in white tea processed by heating withering

The KEGG (Kyoto Encyclopedia of Genes and Genomes) database helped researchers study genes, expression information and metabolite contents as a whole network (31). As the main public database on pathways, KEGG offers integrated metabolic pathway (pathway) queries, covering the metabolism of carbohydrates, nucleotides, amino acids, and the biodegradation of organic matter. It not only provided all possible metabolic pathways but also offered comprehensive annotations of the enzymes that catalyze each step of the reaction. It contained amino acid sequences, links to PDB libraries, etc. It was a powerful tool for conducting metabolic analysis and metabolic network research in organisms.

**Figure 7:**
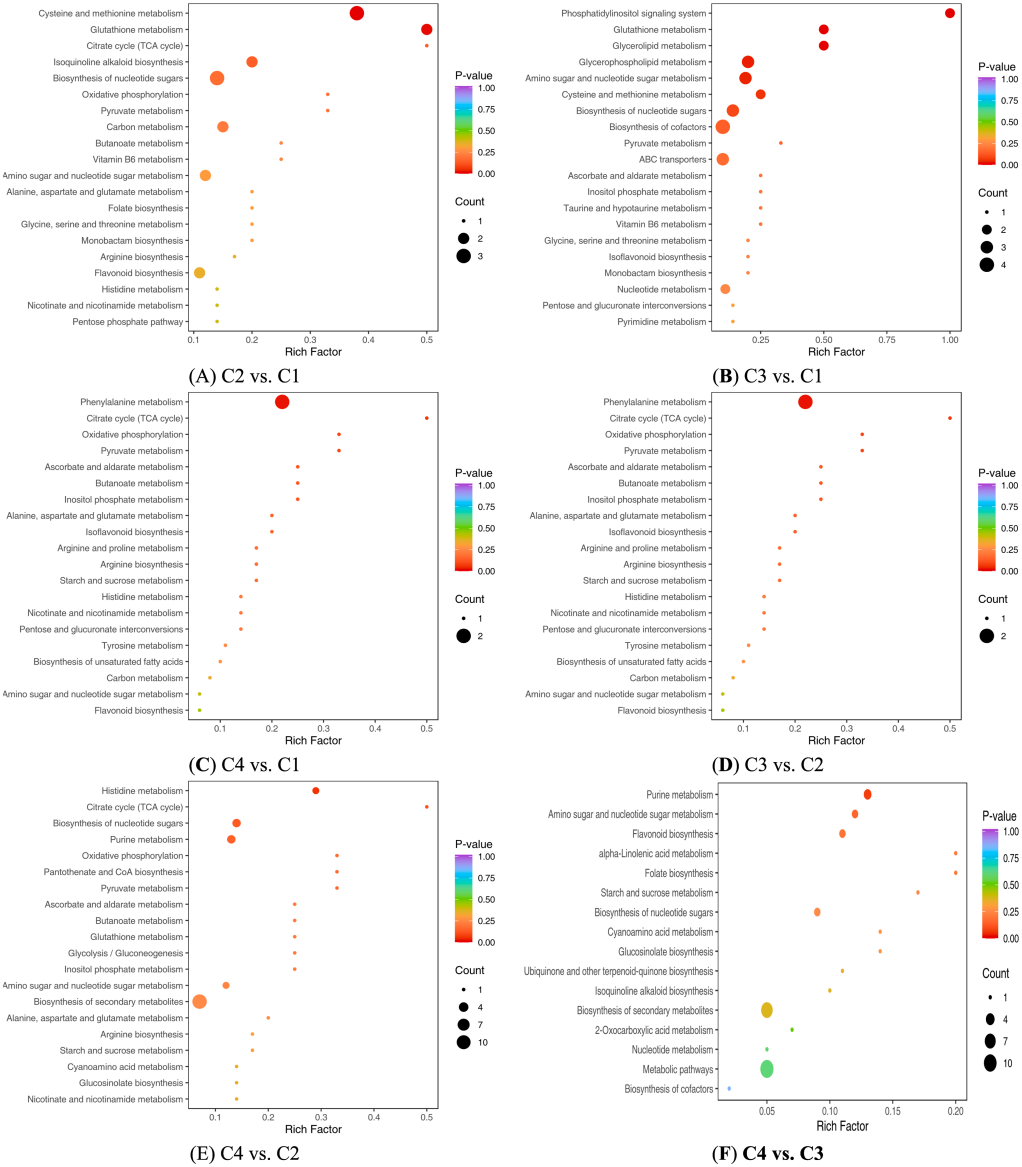
KEGG analysis of four white tea samples with different withering processes. **(A)** KEGG analysis of non-volatile components in C2 vs. C1. **(B)** KEGG analysis of non-volatile components in C3 vs. C1. **(C)** KEGG analysis of non-volatile components in C4 vs. C1 **(D)** KEGG analysis of non-volatile components in C3 vs. A2. **(E)** KEGG analysis of non-volatile components in C4 vs. C2. **(F)** KEGG analysis of non-volatile components in C4 vs. C3. The horizontal axis represented the rich factor corresponding to each path. The vertical axis was the path name (sorted by *p*-value). The color of the point reflected the size of the *p*-value. The redder, the more significant the enrichment. The size of the dots represented the number of differential metabolites enriched.

The main KEGG pathway between C2 and C1 were cysteine and methionine metabolism, glutathione metabolism, isoquinoline alkaloid biosynthesis, amino sugar and nucleotide sugar metabolism, biosynthesis of nucleotide sugars, etc. (Figure 7A). The main KEGG pathway between C3 and C1 were phosphatidylinositol signaling system, glutathione metabolism, isoquinoline alkaloid biosynthesis, amino sugar and nucleotide sugar metabolism, biosynthesis of nucleotide sugars, cysteine and methionine metabolism, biosynthesis of nucleotide sugars, biosynthesis of cofactors, pyruvate metabolism, etc. (Figure 7B). The main KEGG pathway between C4 and C1 were phenylpropanoid biosynthesis, etc. (Figure 7C). The main KEGG pathway between C3 and C2 were phenylpropanoid biosynthesis, etc. (Figure 7D). The main KEGG pathway between C4 and C2 were biosynthesis of secondary metabolites, amino sugar and nucleotide sugar metabolism, biosynthesis of nucleotide sugars, purine metabolism, histidine metabolism, etc. (Figure 7E). The main KEGG pathway between C4 and C3 were purine metabolism, flavonoid biosynthesis, biosynthesis of nucleotide sugars, etc. (Figure 7F). The above research results differ from other research (3, 16), which suggested that the key metabolic pathways involved in the withering of mulberry leaf white tea were starch and sucrose metabolism, as well as glycerophospholipid metabolism etc.

## 4 Conclusion

This study elucidated the effects of various heating withering processes on the taste quality of white tea and their metabolic basis by integrating metabolomics, sensory genomics, and molecular sensory science. Compared with traditional withering at 20 ± 1 °C, hot air, sunlight, and fluorescent lamp withering at 28 ± 1 °C resulted in higher sensory scores. A total of 1,372 metabolites were identified across treatments, including 382 organic acids, 191 amino acids and derivatives, 117 flavonoids, and 49 nucleotides and derivatives, among others. The contents of organic acids, amino acids, flavonoids, and catechins were generally higher in the three heating withering processes than in the control, providing a chemical basis for their superior taste quality. Notably, hot air withering led to higher levels of amino acids and flavonoids compared to fluorescent lamp withering, aligning with its enhanced sensory performance. Metabolite profiling revealed distinct up-regulation and down-regulation patterns in each heating process, particularly under fluorescent lamp withering. Key KEGG pathways enriched in heating treatments included phosphatidylinositol signaling, glutathione metabolism, flavonoid biosynthesis, phenylpropanoid biosynthesis, and amino sugar and nucleotide sugar metabolism.

Future studies should integrate transcriptomic and proteomic approaches to elucidate how photoreceptor signaling pathways and temperature collectively regulate white tea withering. Furthermore, metabolic flux analysis should be employed to quantify the conversion routes and rates of key flavor precursors—such as catechins, amino acids, and fatty acids—under heated withering conditions. By constructing metabolic network models, it will be possible to predict the accumulation of taste-related metabolites under various processing parameters, thereby providing a theoretical foundation for the precision manufacture and quality control of white tea.

## Data Availability

The original contributions presented in the study are included in the article/supplementary material, further inquiries can be directed to the corresponding authors.
